# Three-Dimensional Printed Electrode and Its Novel Applications in Electronic Devices

**DOI:** 10.1038/s41598-018-25861-3

**Published:** 2018-05-09

**Authors:** Chuan Yi Foo, Hong Ngee Lim, Mohd Adzir Mahdi, Mohd Haniff Wahid, Nay Ming Huang

**Affiliations:** 1 Department of Chemistry, Faculty of Science, Universiti Putra Malaysia, 43400 UPM Serdang, Selangor Darul Ehsan Malaysia; 2 Materials Synthesis and Characterization Laboratory, Institute of Advanced Technology, Universiti Putra Malaysia, 43400 UPM Serdang, Selangor Darul Ehsan Malaysia; 3 Wireless and Photonics Network Research Centre, Faculty of Engineering, Universiti Putra Malaysia, 43400 UPM Serdang, Selangor Darul Ehsan Malaysia; 4 New Energy Science & Engineering Programme, University of Xiamen Malaysia, Jalan SunSuria, Bandar SunSuria, 43900 Sepang, Selangor Darul Ehsan Malaysia

## Abstract

Three-dimensional (3D) printing technology provides a novel approach to material fabrication for various applications because of its ability to create low-cost 3D printed platforms. In this study, a printable graphene-based conductive filament was employed to create a range of 3D printed electrodes (3DEs) using a commercial 3D printer. This printing technology provides a simplistic and low-cost approach, which eliminates the need for the *ex-situ* modification and post-treatment of the product. The conductive nature of the 3DEs provides numerous deposition platforms for electrochemical active nanomaterials such as graphene, polypyrrole, and cadmium sulfide, either through electrochemical or physical approaches. To provide proof-of-concept, these 3DEs were physiochemically and electrochemically evaluated and proficiently fabricated into a supercapacitor and photoelectrochemical sensor. The as-fabricated supercapacitor provided a good capacitance performance, with a specific capacitance of 98.37 Fg^−1^. In addition, these 3DEs were fabricated into a photoelectrochemical sensing platform. They had a photocurrent response that exceeded expectations (~724.1 μA) and a lower detection limit (0.05 μM) than an ITO/FTO glass electrode. By subsequently modifying the printing material and electrode architecture, this 3D printing approach could provide a facile and rapid manufacturing process for energy devices based on the conceptual design.

## Introduction

Over the last decade, significant inroads have been made in accelerating the application of advanced two-dimensional (2D) nanomaterials in electronic applications, such as graphene derivatives^1,2^, transition metal oxide^3^, 2D polymer^4^ and molybdenum disulphide^5^. Among these, there has been much interest in graphene materials because of their fascinating electrochemical and physiochemical properties. The high surface area (2630 m^2^ g^−1^)^6^ and strong mechanical properties (~1100 GPa of Young’s modulus)^7^ of graphene have permitted the evolution of the material structure into a three-dimensional (3D) architecture. Research into dimensional evolved graphene materials has been driven by their reinforced properties, which allow the materials to be conventionally applied in various electronic applications such as energy storage and energy conversion devices^8–10^.

Nowadays, the customization and design of complex material structures have been established via the fused deposition modeling (FDM) process of 3D printing. With the aid of thermoplastic polymers such as acrylonitrile-butadiene-styrene (ABS) and polylactic acid (PLA), the potential of 3D printing has been expanded to provide an affordable platform for the production of low-cost 3D components to suit different applications^11^. Research on 3D printed components has recently been conducted with the goal of fabricating a functional electrochemical system because of their rapid manufacturing process and high precision in constructing a complex structure^12^. However, the progress of this printing technique is still particularly focused on the biomedical field such as microfluidic devices^13,14^ and miniaturized electrode materials^15^. In respect to 3D printed energy storage, the first 3D printed micro-supercapacitor utilizing titanium integrated electrodes was established by Zhao *et al*.^16^. In addition to FDM, the reported micro-supercapacitor used another 3D printing technique called Selective Laser Melting (SLM) to construct the metallic Ti_6_Al_4_V electrode. By incorporating polypyrole (Ppy) and a polyvinyl alcohol (PVA) polymer electrolyte through electrodeposition and the solid-state assembly protocol, the fabricated micro-supercapacitor possessed a volumetric capacitance of 2.4 F cm^−3^.

Because of the high material cost of the SLM technique using expensive metal powder^17^ and a laser^18^, the attention on 3D printing has shifted toward ink-jet printing using the same FDM concept. This direct-ink writing protocol utilizes a slurry ink composed of active materials and a polymer binder to construct a 3D electrode array. Using this approach, Zhu *et al*.^19^ fabricated a micro-supercapacitor comprised of a 3D-printed aerogel using an extrudable graphene oxide (GO)-based composite ink with a corresponding gravimetric capacitance of 4.76 F g^−1^ at a current density of 0.4 A g^−1^ in a 3 M KOH aqueous electrolyte. Areir *et al*.^20^ also used a similar printing approach with activated carbon slurries to construct a multilayer electrode material, which exhibited a capacitive performance of 68.7 mF at a scan rate of 20 mV s^−1^. Although these direct-writing protocols hold promise for fabricating electrode materials, in the majority of scenarios, the formulation and optimization of the printable/extrudable ink delays the rapid manufacturing process and limits the choice of material to be printed^14,19,20^. Furthermore, the additional curing or drying process of the ink is non-favorable for creating a freestanding 3D printed electrochemical system. For instance, a 3D microperiodic polyelectrolyte structure constructed using the direct-writing protocol required a coagulation reservoir to solidify the extruded ink prior to its application^21^.

Alongside with the dimensional evolution of graphene, different kinds of printing materials have been established that favor using the printing process for electrochemical systems. Despite the use of slurry ink in the direct-writing protocol, Wei *et al*.^22^ successfully fused graphene with ABS to produce a 3D printable filament (with 5.6 *wt*% graphene loading). Interestingly, this graphene filament could be printed using a relatively low-cost FDM 3D printer, and an electrical conductivity of 1.05 × 10^−3^ S m^−1^ could be achieved. By utilizing a similar printing material, Salvo *et al*.^23^ created the first 3D printed electrocardiogram (ECG) sensor using a commercial 3D printer, in which the electrode was 3D printed and coated with titanium and a gold layer to prevent corrosion and oxidation before being used for ECG measurements. Not only could this fabrication technique be used for ECG measurements, but the electrode structure could also be personalized to address an inadequate contact with the skin, which can be unpleasant and painful for the patient. With evidence of such elegant work reported, the potential of creating a low-cost and functional electrochemical platform with the aid of a conventional 3D printing technique and conductive thermoplastic filament has been identified.

The main purpose of this paper is to develop a supercapacitor and a PEC sensor using FDM technology of 3D printing technique. It is undeniable that FDM technology has been introduced in the early 1990s, and various studies had reported using this technology. However, the FDM technique has evolved and modified for the past decade to suit the individual needs of different industry. The recent review on the application of FDM in various electronic devices has proven that this innovative approach can greatly enhance the development in electrochemical energy storage systems and other related applications^8–10^. The novelty of this study can be divided into 2 categories, which are (1) development of a supercapacitor and a PEC sensor device, and (2) fabrication of a three-dimensional electrode (3DE) using a 3D printer technique. To the best of our knowledge, there is no investigation of supercapacitor and PEC sensor fabricated using a 3D printed electrode. The commercial electrode materials for supercapacitor and PEC research are rigid substrates such ITO/FTO glass, glassy carbon and conductive fabric materials^24,25^. These rigid substrates are expensive and material processing is tedious when fabricating a functional electronic device. For instance, the customization of an electrode using an ITO/FTO glass require a specialized cutter to cut out the desired shape for an electrochemical study. This problem also occurs in other metal substrates such as nickel foam and platinum plate. In comparison, 3D printing can provide a rapid and economic approach in creating a customized electrode structure for different research applications. The 3DE can be print according to the desire shape and size, which prevents wastage in materials processing.

The development of a green electronic device with low-manufacturing cost, rapid production, good safety and high reliability is urgently in demand to address environment pollutions in today’s society. Biodegradable carbon-based electrode (3DE) is an alternative electrode material that is environment and user friendly^26^. Moreover, this carbon-based electrode, with a photocurrent response of 3 orders of magnitude higher than those rigid glass and conductive fabric substrates, can achieve good PEC performance at a lower cost^27^. This can reduce the processing cost and environment pollution caused by electronic wastes.

In this study, we report a FDM 3D printing process used to produce a functional electrode for electrochemical devices. This fabrication process utilizes a conductive graphene filament and a commercial Alpha 3D printer to print out the electrode. It provides a rapid and cost-effective approach to producing a customized architecture, compared to the direct-writing protocol and other 3D printing methods. Ppy was electrodeposited on the electrode surface as an electroactive material, and a symmetrical solid-state supercapacitor was demonstrated using a PVA-KOH polymer electrolyte. Furthermore, for the first time, the utilization of a 3D printed electrode for a photoelectrochemical (PEC) sensor, as an alternative to the commonly used optical transparent electrode, was also being demonstrated in this study. Overall, this paper will trigger more research in the fields of supercapacitor and PEC, leading to a venture on the FDM technology by utilizing a 3D printing technique, aiming to produce components with a more complex geometry according to computer designs and/or wearable electronics devices that are biodegradable.

## Results and Discussion

### Physiochemical properties of three-dimensional printed electrode (3DE)

**Figure 1:**
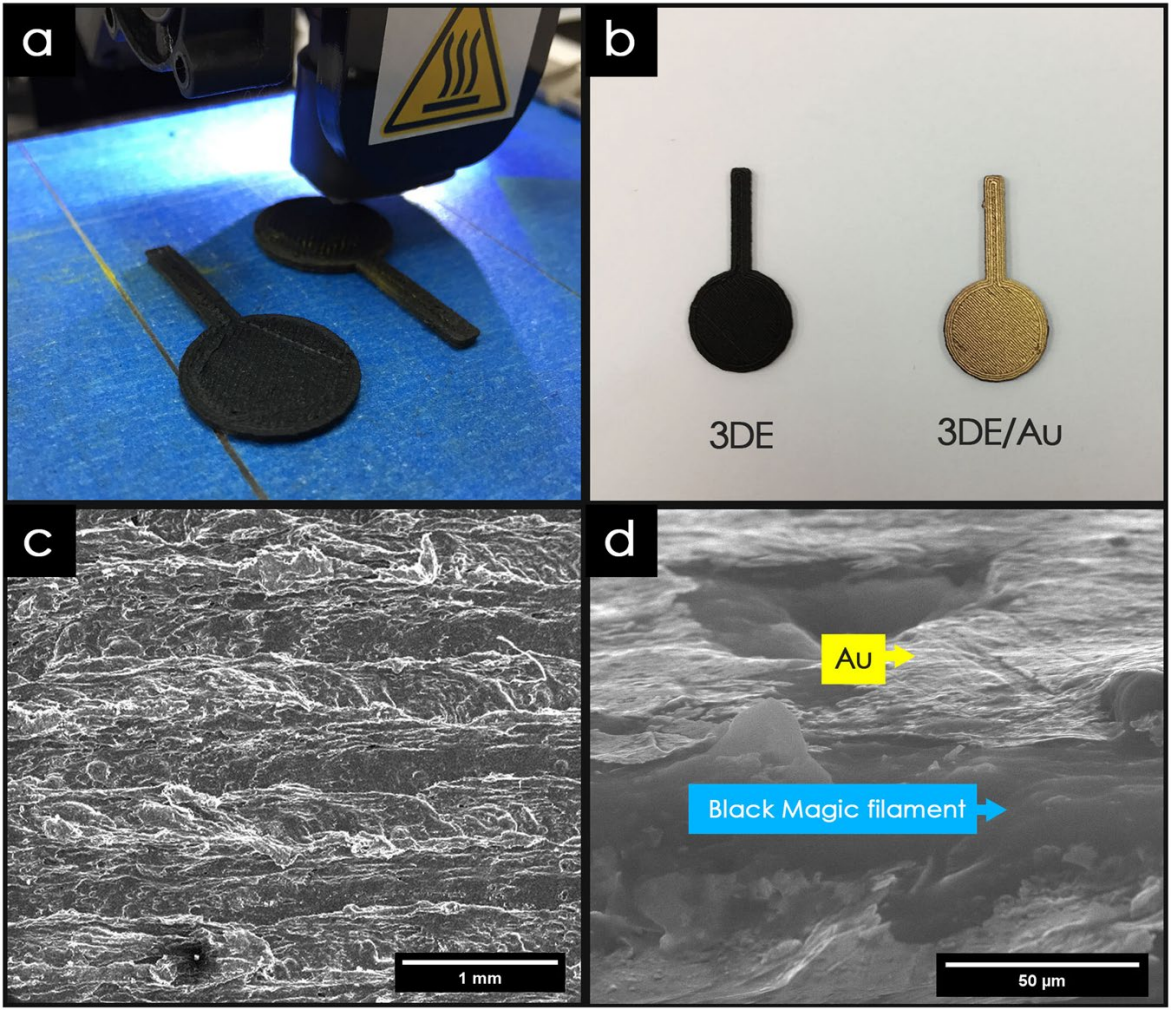
Physiochemical characterization. (**a**) Optical image of 3D printing process, (**b**) 3D printed electrode used throughout study. (**c**) FESEM image of 3DE/Au electrode, and (**d**) corresponding magnified cross-sectional area.

The fabrication process for the 3DE utilizing the FDM 3D printing technique provides a time efficient and low-cost approach to mass producing electrode materials. Compared to commercial electrodes such as copper, aluminum, and carbon electrodes, the architecture and surface area of the 3DE can be easily customized to suit a particular application, as shown in Fig. 1a. The printing process for the 3DE was fully automated, with a high degree of precision (with 0.4 mm nozzle size), which made it possible to complete the printing process for eight 8 electrodes in just 30 min. Furthermore, using a conductive filament as the printing material can facilitate the application of 3DEs in electronic fabrications, which would allow the printed thermoplastic material to conduct electricity without any post-treatment. The detailed characterization of the Black Magic filament was not carried out because it has been available in the market as a commercial end product^28,29^. The graphene content in the commercial conductive filament (from Black Magic) is around 8%, and the chemical composition of the filament consists of just carbon and oxygen, according to the investigation of the physiochemical properties of the same conductive filament conducted by Foster *et al*.^28^. Although the 3DE could conduct electricity, the surface resistance was too high, which hindered its application in electronic devices. Therefore, a thin layer of gold was sputtered onto the surface of the 3DE to reduce the surface resistivity (Fig. 1b).

The surface uniformity of the gold sputtered 3DE (3DE/Au) was investigated *via* a field emission scanning electron microscope (FESEM) analysis. The surface of the 3DE/Au is shown in Fig. 1c, where it is clearly seen that the extruded filament is around 0.4 mm and printed in a uniform array. In the cross section of the 3DE/Au shown in Fig. 1d, a thin layer of gold can be noticed on the electrode surface with a lighter contrast. Furthermore, polymer nanowires and several crystalline binders were embedded within the Black Magic filament. The chemical compositions of the 3DE/Au and 3DE were determined *via* energy dispersive X-ray (EDX) analyses within ESI Figure S1, which shows that significant carbon and oxygen peaks predominate throughout the spectra. This indicates that the electrical conductivity of Black Magic filament was mainly contributed by the of organic-based graphene materials. The presence of gold on the 3DE/Au could also be detected in the EDX spectra.

### Electrodeposition of Ppy/rGO nanocomposite on 3DE/Au electrode

**Figure 2:**
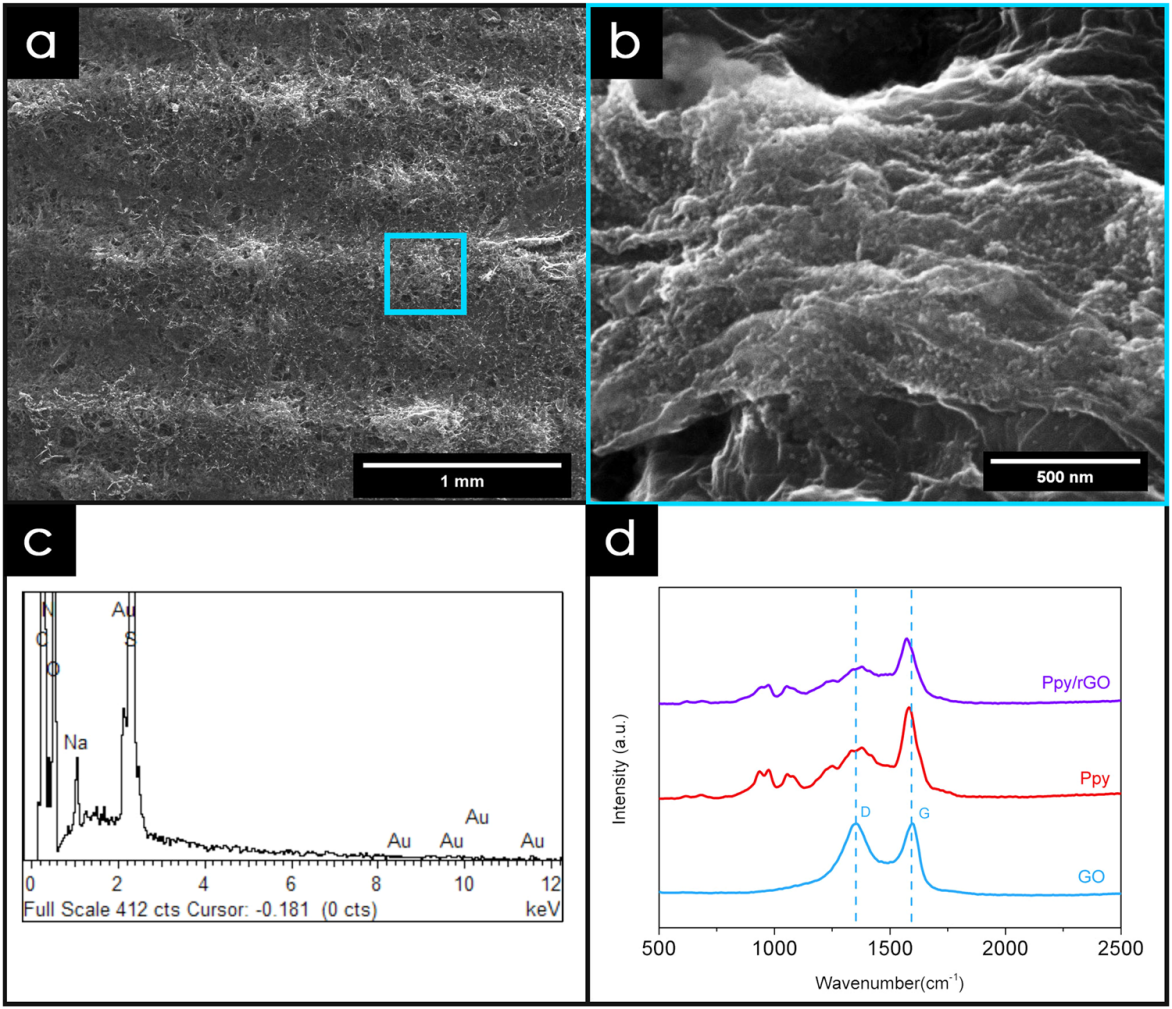
FESEM image of (**a**) Ppy/rGO nanocomposite on the electrode surface and (**b**) its corresponding magnified image. Corresponding (**c**) EDX and (**d**) Raman analysis of Ppy/rGO nanocomposite.

To the best of our knowledge, this is the first time that 3DE and 3DE/Au were used as novel electrodes for the deposition of a Ppy/rGO nanocomposite *via* the electrodeposition method. From the FESEM images (Fig. 2a), we can clearly see that the surface of the 3DE/Au was fully covered with Ppy/rGO, with noticeable pores scattered across the electrode surface. Moreover, the Ppy nanoparticles were found to be distributed on the rGO surface through the magnified FESEM image (Fig. 2b). An EDX analysis was carried out to justify the presence of the Ppy/rGO nanocomposites on the 3DE/Au surface, as shown in Fig. 2c. It is clear that carbon, nitrogen, and oxygen are the most predominant peaks, indicating that the deposited nanocomposites were Ppy/rGO. Furthermore, an intense peak for Au was observed, indicating that a thin layer of Ppy/rGO nanocomposites was deposited on the 3DE/Au electrode, which allowed the gold from the electrode surface to be identified within a single spectrum.

The Ppy/rGO deposition process was initiated by the application of an oxidation potential (+0.8 V vs. Ag/AgCl), wherein the pyrrole monomers were electrochemically oxidized to start the polymerization. The neutral monomer was oxidized to form radical cations with a delocalized radical state and later formed a complex with the pTS anions through the electrostatic charge attraction. The coupling of the pyrrole monomers formed a larger dimer, which was then immediately reoxidised to form a cation. The propagation of the polymer chains continued as more newly form radical cations were introduced to the dimeric chain, and eventually formed the Ppy nanocomposites. Interestingly, the presence of GO with a negatively charge surface provided an alternative anchoring site for the pyrrole monomer. The GO was then subsequently reduced to rGO upon the release of electrons from the pyrrole radical cations. The free electrons released from the formation of radical cations reduced the GO into rGO, and the polypyrrole nanoparticles were attached on the rGO surface through π-π interaction, hydrogen bonding, and Van der Waal forces^30,31^.

To validate the interaction between the Ppy and rGO, Raman spectroscopy was carried out, and the spectra are shown in Fig. 2d. Two significant GO peaks can be observed at 1356.27 cm^−1^ and 1597.67 cm^−1^, which are assigned to the D-band and G-band, respectively. The D-band of GO is related to the surface defect of the hexagonal lattice, whereas the G-band corresponds to the sp^2^ carbon bond stretching^32,33^. For pure Ppy, the significant peaks observed at 1582.52 cm^−1^ and 1378.14 cm^−1^ correspond to the C=C backbone stretching and ring stretching mode of Ppy, respectively. Moreover, the C-H bond deformation peak can be found at 1056.50 cm^−1^ in the same spectrum. In addition, the two small peaks located at 971.40 cm^−1^ and 934.17 cm^−1^ correspond to the ring deformation of the radical cation (polaron) and bication (bipolaron), respectively^34,35^. The results of an evaluation of the significant peaks for the Ppy/rGO nanocomposites are represented in Table 1, and are compared to those for the GO and pure Ppy. Overall, it is clear that the characteristic peaks of pure Ppy can be found on the Raman spectrum of the Ppy/rGO nanocomposites, which verified the presence of Ppy within the nanocomposites. Moreover, by taking into account the D/G band ratio (I_D_/I_G_), the atomic sp^3^/sp^2^ carbon ratio can be calculated to measure the graphitic disorder of the GO material^36^. Interestingly, the calculated I_D_/I_G_ ratio for the Ppy/rGO nanocomposites (0.80) was lower than that for pure GO (0.99), which showed that the graphitic disorder of the Ppy/rGO was less than that of pure GO. In addition, the shifting of the G-band from 1597.67 cm^−1^ to 1575.40 cm^−1^ further proved that the GO was rGO during the electrodeposition process^37,38^.

**Table 1:** Raman analysis of GO, pure Ppy and ppy/rGO nanocomposites.

Component	Wavenumber (cm^−1^)	Assignment	I_D_/I_G_
GO	1356.27	D-band	0.99
	1597.67	G-band	
Ppy	934.17	Polaron	0.70
	971.49	Bipolaron	
	1056.50	C-H deformation	
	1378.14	Ring stretching	
	1582.52	C=C stretching	
Ppy/rGO	937.49	Polaron	0.80
	974.89	Bipolaron	
	1057.34	C-H deformation	
	1377.00	Ring stretching	
	1573.40	C=C stretching	

### 3DE/Au based solid-state supercapacitor

The electrical function of the 3DE made using different materials was shown in ESI Figure S2. Ppy/rGO nanocomposite was electrodeposited on top of the 3DE made by Black Magic filament and ordinary PLA filament. Due to the electrical insulator nature of ordinary PLA filament, a thin layer of gold was sputtered on top of the 3DE made from PLA filaments before electrodeposition. Since the ordinary PLA filament has no electrical conductivity, the Ppy/rGO was deposited only on the thin layer of sputtered gold on the electrode surface. The CV profile of the 3DE made by ordinary PLA filament was fully contributed by the Ppy/rGO on the thin gold layer. Due to the limited reactive surface area for active material deposition, the CV performance of 3DE made by ordinary PLA filament was very small. On the other hand, the area under the CV curve of the 3DE made from Black Magic filament was significantly larger than the 3DE made from ordinary PLA filament. This is because the conductive nature of the Black Magic filament increases the reactive surface area of the 3DE for electrodeposition of Ppy/rGO nanocomposite.

**Figure 3:**
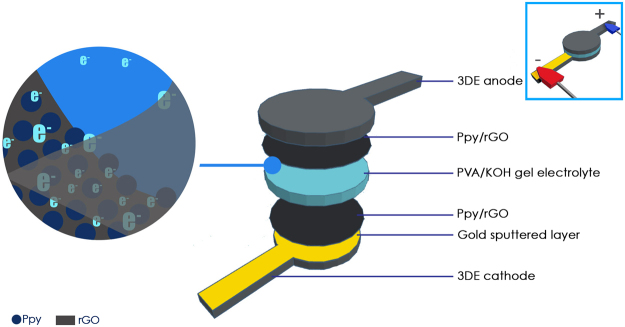
Schematic illustration of solid-state supercapacitor fabrication.

**Figure 4:**
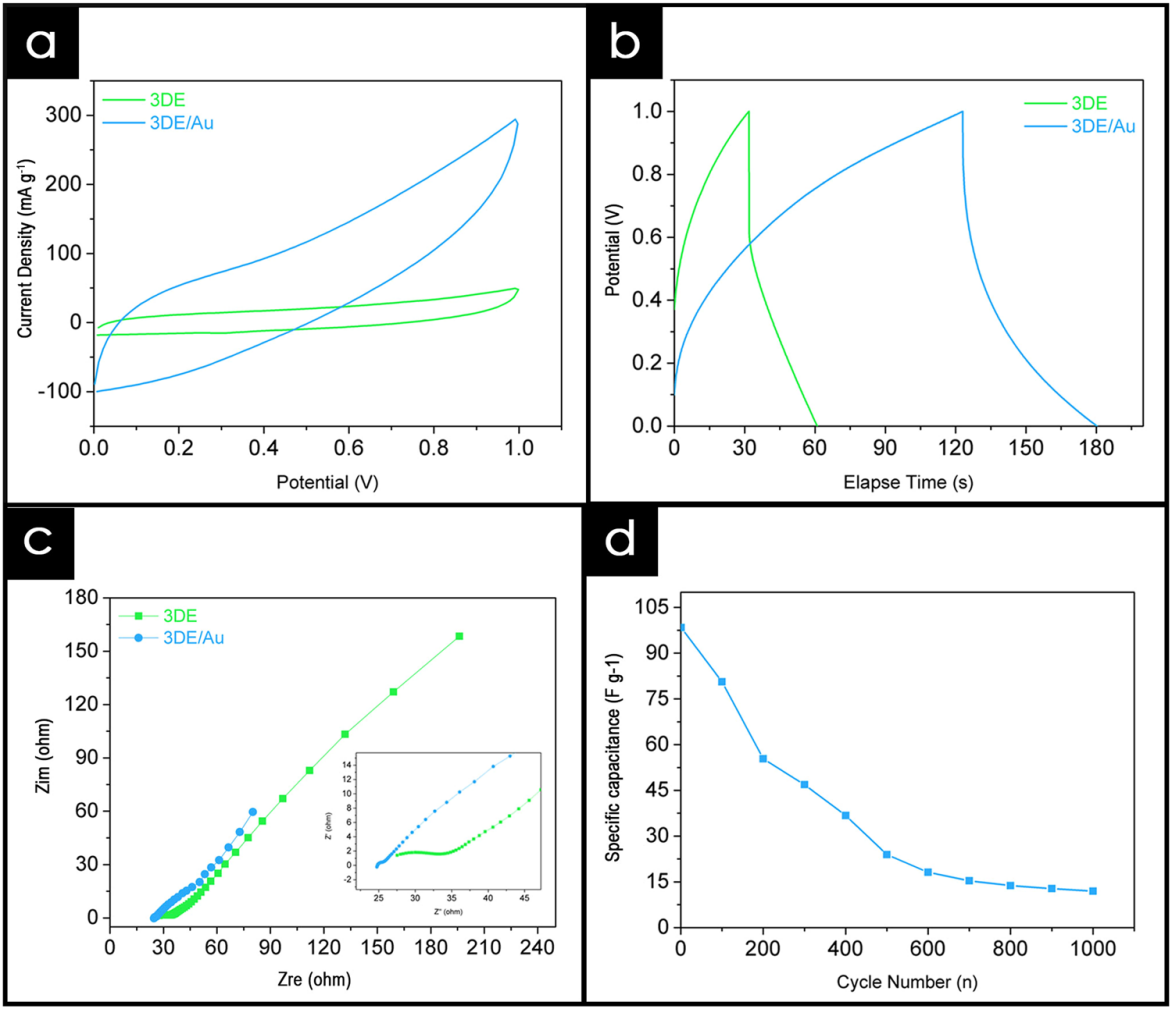
Electrochemical performance of 3DE/Au-based supercapacitor. (**a**) Cyclic voltammogram analysis over 1.0 V potential range at scan rate of 50 mV s^−1^, (**b**) corresponding galvanostatic charge/discharge profile at current density of 0.5 Ag^−1^, (**c**) Nyquist plot, and (**d**) cyclic stability profile of as-fabricated supercapacitor.

A fully freestanding supercapacitor was developed utilizing two Ppy/rGO decorated 3DE/Au electrodes, with a PVA-KOH gel electrolyte sandwiched between them, as shown in Fig. 3. The potential of this novel 3DE/Au electrode was evaluated through the electrochemical performance of the as-fabricated solid-state supercapacitor. For comparison, Ppy/rGO decorated 3DE electrodes were also tested under the same procedure. After the creation of the solid-state supercapacitors, cyclic voltammetric (CV) analyses were carried out over a potential range of +0.0 V to 1.0 V, at a scan rate of 50 mV s^−1^. The CV profile (Fig. 4a) shows the general capacitive property of the supercapacitor, and the area under the curve is indicative of the capacitance of the system. Overall, the 3DE/Au-based supercapacitor had a larger current response than the 3DE, because the presence of the gold layer facilitated the electrical conductivity and ionic transport during the operation of the charge storage mechanism. Next, the galvanostatic charge/discharge (GCD) cycling of the solid-state supercapacitors was carried out to evaluate their specific capacitance, C_sp_. A typical shark fin GCD profile was found for the 3DE/Au-based supercapacitor (Fig. 4b), indicating good charge/discharge properties with a minor IR drop. The specific capacitance was calculated to be 98.37 Fg^−1^ at a current density of 0.5 Ag^−1^, which was approximately three times that of the 3DE-based supercapacitor (32.46 Fg^−1^). The Nyquist plots for both supercapacitors in a frequency range of 0.1 Hz to 300 kHz are illustrated in Fig. 4c. A small semicircle can be noticed in the high-frequency region of the 3DE/Au-based supercapacitor, indicating a small charge transfer resistance (R_ct_) at the electrode/electrolyte interface. Furthermore, the equivalent series resistance (ESR), including the contact resistance at the active/current collector interface, ionic resistance of the electrolyte, and intrinsic resistance of the substrate, could be deduced from the intercept at the real axis (Z_re_). The presence of the gold layer reduced the R_ct_ value of the 3DE from 15.4 Ω to 1.3 Ω, as well as the ESR value (from 27.5 Ω to 24.7 Ω). These EIS results further demonstrated the enhanced electrical conductivity and ionic mobility of the 3DE/Au-based supercapacitor. In addition, the 3DE/Au-based supercapacitor exhibited good cycling stability (Fig. 4d) over the first 100 charge/discharge cycles (a capacitance retention of 81.94%). However, because of the organic-based 3DE/Au electrodes and evaporation of the electrolyte from the PVA-KOH gel matrix, the capacitance retention drastically dropped to 12.15% after 1000 charge and discharge cycles. The Ppy/rGO layer on the 3DE/Au surface shriveled (ESI Figure S3) due to insufficient electrolyte support from the PVA-KOH gel layer. Consequently, the overall reactive surface area of the electrode was greatly reduced, resulting in poor capacitance performances.

### 3DE/Au-based photoelectrochemical sensor utilizing cadmium sulfide nanoparticles

The application of 2D and 3D nanomaterials in electronic applications has received much interest from a profusion of material chemists studying the exploration and exploitation of their distinctive characteristics. We next discuss how this 3DE/Au electrode was utilized for the photoelectrochemical (PEC) sensing of copper ions using cadmium sulfide as an active semiconductor^39,40^. The most common current collector for a PEC sensor is indium-doped tin oxide (ITO) or fluorine-doped tin oxide (FTO) glass^25,41^. These optically transparent electrode materials provide high optical transmittance values, which allow a greater photoelectrical response compared to a glassy carbon electrode (GCE)^42^. As outlined in the introduction, one of the objective of this research was to find a possible substitute for the currently available electrode materials. We therefore investigated the potential application of the 3DE/Au as an electrode material for a PEC sensor with the aim of providing important insights into its photoelectrochemical properties.

**Figure 5:**
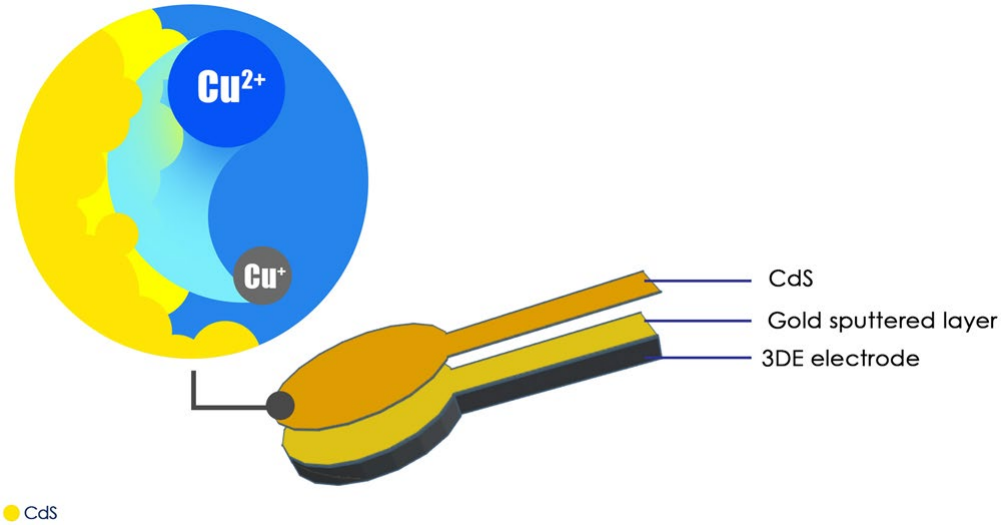
Schematic illustration of PEC sensor using 3DE/Au electrode.

**Figure 6:**
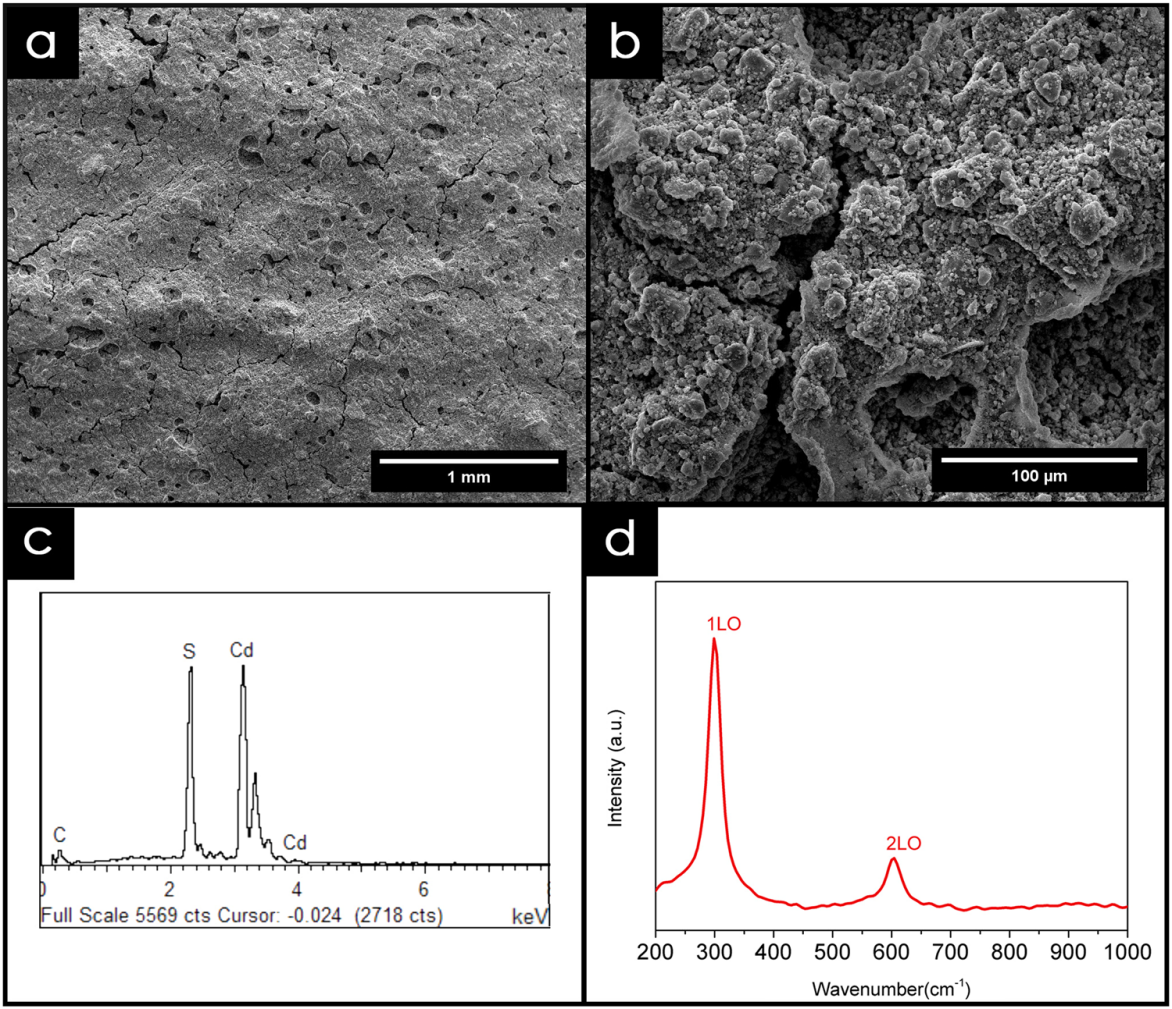
Characterization of CdS nanoparticles. (**a**) FESEM image and (**b**) magnified image of CdS deposited 3DE/Au electrode surface. Corresponding (**c**) EDX and (**d**) Raman analysis of the CdS nanoparticles.

Initially, the cadmium sulfide nanoparticles were synthesized through a facile solvothermal method with cadmium acetate and thiourea as the precursor reagents. Upon application, Nafion was mixed with the as-synthesized CdS nanoparticles as a binder to produce a yellowish paste. The paste was then applied to the surfaces of both the 3DE/Au and 3DE electrodes to form PEC sensors (Fig. 5). The FESEM images of the electrode surfaces are shown in Fig. 6a. The high mechanical stability of Nafion acted as an excellent binder to hold the CdS nanoparticles on the electrode surface^43^. The small cluster of CdS nanoparticles found on the magnified electrode surface (Fig. 6b) was a result of the self-agglomeration of the nanoparticles due to their high surface energy nature^44^. However, this phenomenon had no effect on the chemical composition or performance of the as-fabricated PEC sensor. This could be verified by the significant profile from the EDX and Raman analyses (Fig. 6c,d). Despite the electrodeposition of the Ppy/rGO nanocomposite, the thickness of the CdS layer was not easily manipulated by the doctor blade application. Therefore, only Cd and S peaks could be observed to be dominant in the EDX profile, with a small noticeable peak of C from the Nafion binder. This also showed that the nanoparticles deposited on the electrode surface were purely CdS with no contamination. The Raman analysis further showed the purity of the CdS nanoparticles, which had two distinctive peaks at 299.4 cm^−1^ and 601.1 cm^−1^, corresponding to the first-order (1LO) and second-order (2LO) longitudinal optical phonon modes of CdS, respectively^27^.

### Photoelectrochemical sensing of copper ion

**Figure 7:**
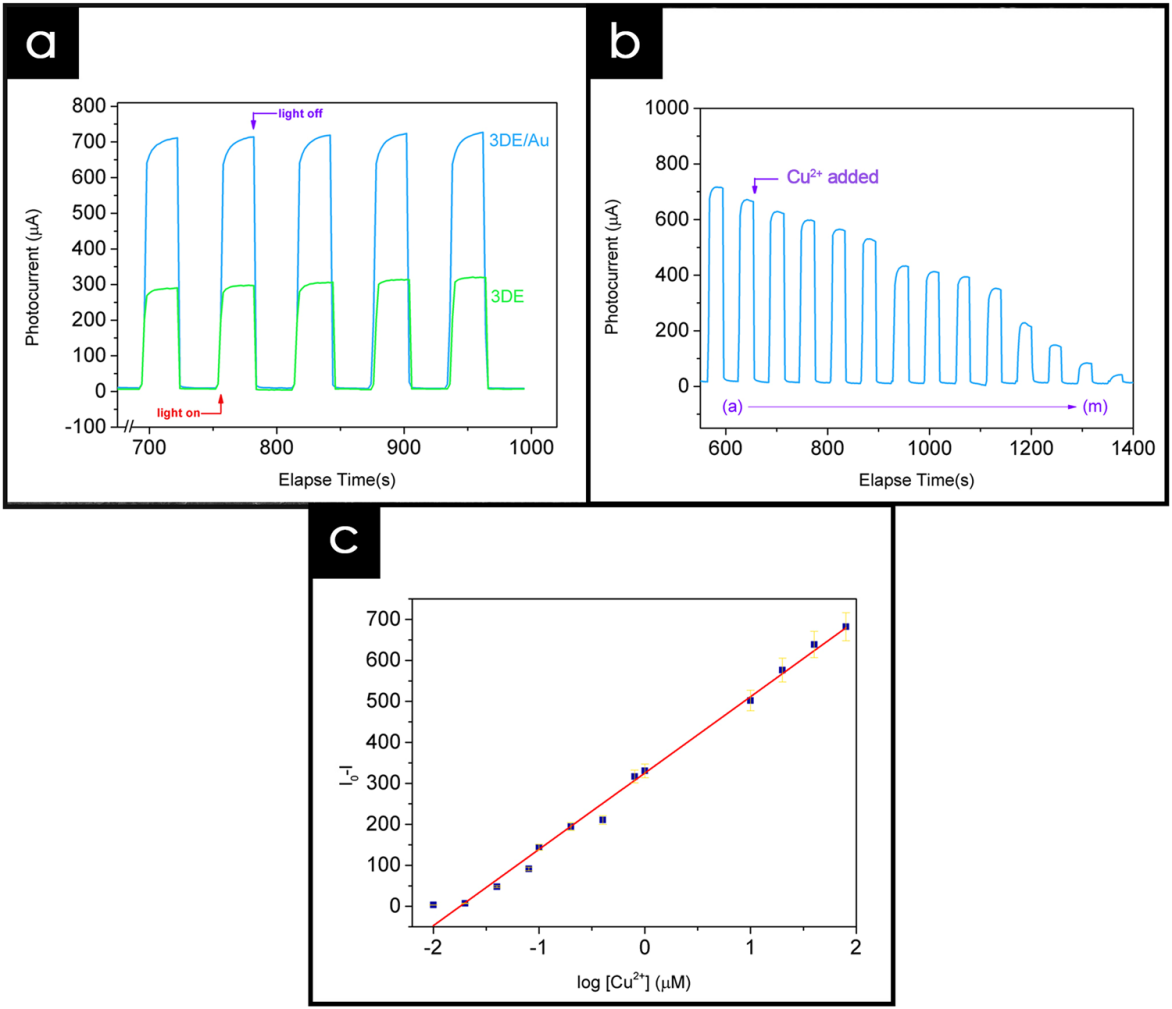
Photoelectrochemical performance. (**a**) Time-based photocurrent response of 3DE and 3DE/Au-based PEC sensor, measured in KCl:TEA electrolyte at bias potential of 0.1 V. (**b**) Effect of Cu^2+^ on the photocurrent response of 3DE/Au-based PEC sensor as concentration increases from 0.01 μM to 80 μM. (**c**) Linear relationship between the Cu^2+^ concentration and photocurrent response, where I_0_ and I represent photocurrent intensities without and with the presence of Cu^2+^, respectively.

The photoelectrochemical properties of the 3DE/Au- and 3DE-based PEC sensors were evaluated prior to their application as a copper sensing platform. Surprisingly, the PEC performance of the as-fabricated sensor showed a remarkably good result in response to simulated light. Although the electrode material had an opaque structure, the conductive nature of the Black Magic filament and Au layer made it possible to transport the photo-excited electrons from the CdS nanoparticles and convert them into an electrical signal. As shown in Fig. 7a, the photocurrent intensity of the 3DE/Au-based PEC is apparently higher than that of the 3DE-based sensor, with values of 724.1 μA and 309.1 μA, respectively. The photocurrent generated was tremendously high compared to those of the previously reported CdS-modified electrodes^27^ and was reproducible after several on-off light illumination cycles. In addition, the presence of the Au layer in the 3DE/Au electrode enhanced the photocurrent stability throughout the illumination cycles, as shown in ESI Figure S4. Upon light illumination, the photo-excited electrons transferred from the valance band (VB) to the conduction band (CB) of the CdS nanoparticles, and then to the current collector. Because of the highly conductive Au coating on the 3DE/Au surface, the electron mobility was greatly enhanced and provided a rapid and stable photocurrent response compared to the bare 3DE. The high and stable photocurrent response of the 3DE/Au electrode could thus provide a possible alternative to an ITO/FTO glass electrode in a PEC sensing platform.

By utilizing the competitive binding effect between the CdS and Cu^2+^ ion, the as-fabricated 3DE/Au-based PEC sensor could detect a trace amount of Cu^2+^ ions in the electrolyte^45^. In brief, the electron transport between the CdS nanoparticles and 3DE/Au surface was interrupt by the presence of Cu^2+^ ions. The Cu^2+^ was reduced to Cu^+^ on the electrode surface by accepting the photo-excited electrons from the CdS nanoparticles. As a consequence, the formation of Cu_*x*_S (*x* = 1, 2), which subsequently replaced the Cd^2+^ on the electrode surface, could occur during the light illumination process. Because of the reduced amount of CdS on the electrode surface, the photocurrent response of the 3DE/Au-based sensor periodically dropped in the electrolyte containing copper ions. Figure 7b shows that the reduced photocurrent response was highly dependent on the Cu^2+^ concentration, as the concentration increased from 0.01 μM to 80.0 μM. In order to obtain the limit of detection (LOD) of the 3DE/Au-based PEC sensor, the change in photocurrent intensity (I_0_ − I) was plotted against the Cu^2+^ concentration (Fig. 7c), where I_0_ and I are the photocurrent intensities without and with the presence of Cu^2+^, respectively. An excellent linear relationship of R^2^ = 0.99081 was shown, and the calculated detection limit was 0.05 μM, using 3σ/S, where σ is the standard deviation of a blank sample, and S is the slope of the linear calibration curve.

## Conclusion

For the first time, a proof-of-concept is reported involving the application of a graphene-based conductive filament that was 3D printed to a practical electrochemical platform. The physiochemical and electrochemical properties of the 3D printed electrode (3DE) were investigated to determine the maximum potential as an alternative electrode material. With the aid of a graphene-based conductive filament to construct the electrode architecture, a variety of nanomaterials can now be deposited on an electrode surface either through electrochemical or physical approaches. Ultimately, these electrodes could be fabricated into a solid-state supercapacitor with a promising capacitive performance and stable cycling stability of up to 100 charge/discharge cycles. Furthermore, the 3DE was fabricated into a photoelectrochemical sensing platform, in which the photocurrent response exceeded expectations and the detection limit was lower than that of an ITO/FTO glass electrode.

We believe that these 3DEs can provide a low-cost and environmentally friendly approach to the fabrication of electronic devices, whereby no expensive metallic current collector is required, which will reduce the overall manufacturing cost and time. In addition, the introduction of the 3D printing technique with conductive filament in device fabrication could provide an infinite number of architectures and sizes, without additional modification or *ex-situ* post-treatment. In short, the findings of this research utilizing 3D printing in device fabrication could be a building block for the next generation energy architecture.

## Experimental

### Materials

Graphite powder was purchased from Ashbury Graphite Mills Inc. (code no. 3061). Common chemicals such as iron (III) chloride (FeCl_3_, 98%), potassium permanganate (KMnO_4_, 99%), sodium *p*-toluenesulfonate (NapTS, 70%), sulfuric acid (H_2_SO_4_, 95–98%), phosphoric acid (H_3_PO_4_, 85%), ethyl alcohol (C_2_H_6_O), hydrogen peroxide (H_2_O_2_, 30%), hydrogen chloride (HCl, 37%), potassium chloride (KCl), cadmium acetate dihydrate (CH_3_COO_2_Cd.2H_2_O, 97%), and thiourea (NH_2_CSNH_2_, 96%) were procured from Merck. Triethanolamine (TEA) was obtained from Sigma Chemical, Co. N-butylamine (CH_3_(CH_2_)_3_NH_2_) and pyrrole monomer (99%) were purchased from Acros Organics. Pyrrole monomer was stored at 0 °C and distilled prior to use. Conductive filament was purchased from Black Magic. Double distilled water was used throughout the experiments.

### Fabrication of Three-dimensional Printed Electrode (3DE)

The electrode structure was drawn *via* Tinkercad, to create a circular disk shape with a range of thicknesses and a diameter of 17 mm. The 3D electrode model was exported in the *stl* format and printed using a Designex3D Alpha series printer with a direct drive extruder (0.4 mm nozzle size) at a temperature of 220 °C. The commercial conductive filament from Black Magic was used as the printing material, which has a labeled conductivity of 2.13 S cm^−1^. The printing speed was set at 30 mm s^−1^ with 110% filament flow. Each of the electrodes was printed with an elongated strip to facilitate the crocodile clip connection. A layer of gold was sputtered on the surface of the 3D printed electrode, and the fabricated electrode was denoted as 3DE/Au. For comparison, a bare electrode without the sputtered gold was denoted as 3DE.

### Preparation of Ppy/rGO nanocomposite on 3DE/Au electrode

The fabricated 3DE/Au (with a diameter of 17 mm and thickness of 1 mm) was used as the current collector and working electrode in the supercapacitor fabrication. The polypyrrole/reduced graphene oxide (Ppy/rGO) nanocomposites were deposited on the 3DE/Au surface *via* an *in-situ* electrochemical polymerization method. First, 0.1 M of pyrrole monomer and 0.1 M of NapTS were dispersed in a 1 mg/mL GO solution under continuous stirring for 5 min to ensure homogeneity. The formation of Ppy nanoparticles was initiated by the introduction of 0.1 M FeCl_3_ into the precursor mixture, which was vigorously stirred for another 5 min to obtain a Ppy/GO mixture. The electrodeposition of the Ppy/rGO nanocomposites was carried out at a constant potential of +0.8 V using a potentiostat/galvanostat at room temperature for 900 s. The deposited electrode was rinsed thoroughly with de-ionized water to remove any residue and impurities. During the electrodeposition process, a platinum rod and Ag/AgCl were used as the counter and reference electrodes, respectively. For comparison, the Ppy/rGO nanocomposite was also deposited on a bare 3DE without gold sputtering.

### Fabrication of solid-state supercapacitor

The 3DE/Au/PRG electrodes were dried at 60 °C overnight prior to the fabrication of the solid-state supercapacitor. A PVA-KOH gel electrolyte was prepared as the dielectric material. First, 3.0 M of KOH was added to the 10* wt*% PVA solution. The mixture was heated at 90 °C under continuous stirring to obtain a clear PVA-KOH solution. The solution was left under room temperature for 15 min until it turned into a viscous slurry. In order to assemble the solid-state supercapacitor, the prepared PVA-KOH gel electrolyte was slowly poured onto two 3DE/Au/PRG electrodes and air-dried at room temperature for 3 h. Then, the two electrodes were pressed together before the complete solidification of the gel electrolyte, which allowed the PVA-KOH gel on each electrode to fuse into one single separating layer to form an integrated device.

### Preparation of CdS nanocomposite on 3DE/Au electrode

The CdS nanoparticles were synthesized *via* a solvothermal method. First, 15 mmol of thiourea was added to 50 mL of n-buthylamine, followed by 5 mmol of cadmium acetate under continuous stirring to obtain a clear solution. The precursor solution was then transferred into a Teflon-lined stainless steel autoclave, and kept at 100 °C for 24 h. After the reaction was completed, the autoclave was allowed to cool, and the product was centrifuged with ethanol and deionized water at 8500 rpm for 15 min. The yellow precipitates from the centrifuge were collected and kept at 50 °C to remove excess moisture. During their application, the CdS nanoparticles were mixed with Nafion in a ratio of 1:0.5, creating a yellowish paste. The paste was then doctor bladed onto the 3DE/Au surface.

### Characterization

The surface morphology and element analysis results were obtained using a field emission scanning electron microscope (FESEM, FEI Quanta SEM Model 400 F) equipped with an energy dispersive X-ray (EDX) accessory. Raman spectroscopy was conducted using a WITec Alpha 300 R spectrometer with an argon laser (531.79 nm excitation wavelength). Voltammetric measurements were conducted using a Princeton potentialstat/galvanostat controlled by Versa Studio software. The electrochemical characterizations of the as-fabricated electrodes were performed utilizing a three-electrode setup with a 3DE as the working electrode, platinum rod as the counter electrode, and Ag/AgCl as the reference electrode.

The electrochemical impedance study (EIS) was conducted at a frequency range of 0.01 Hz to 30 kHz at an open circuit potential with an AC perturbation of 10 mV. The galvanostatic charge-discharge profile was used to calculate the specific capacitance (C_sp_) of the as-assembled solid-state supercapacitor, using the following equation:

1 $${C}_{sp}=\frac{I{\rm{\Delta }}t}{m{\rm{\Delta }}V}\,(F{g}^{-1})$$

where *I* is the constant discharge current (A), *Δt* represents the discharging time (s), *m* indicates the mass of the active materials (g), and *ΔV* represents the potential range (V).

The photoelectrochemical studies were performed utilizing the three-electrode system, with a 150-W xenon arc lamp with an AM 1.5 G filter solar simulator as the visible light source. A 0.1 M KCl solution was used as the supporting electrolyte, and 0.5 M TEA was added as the sacrificial electron donor. For the Cu^2+^ ion detection, different concentrations of Cu^2+^ (0.01–80.0 μM) were introduced into the electrolyte solution, and the photocurrent responses were recorded using a chronoamperometry technique.

### Data availability statement

All dataset generated during and/or analyzed during the current study are available from the corresponding authors on reasonable request.

## Electronic supplementary material

Supplementary Information
