# Supplementary material for: Three-Dimensional Printed Electrode and Its Novel Applications in Electronic Devices
Source: Sci Rep. 2018 May 9;8:7399. doi: 10.1038/s41598-018-25861-3 (PMC5943534; doi:10.1038/s41598-018-25861-3)
Supplement: Supplementary file 1 — Supplementary Information [file 41598_2018_25861_MOESM1_ESM.doc]

**Three-Dimensional Printed Electrode and Its Novel Applications in Electronic Devices**

Chuan Yi Foo^1^, Hong Ngee Lim^1,2*^, Mohd Adzir Mahdi^3^, Mohd Haniff Wahid^1^ and Nay Ming Huang^4*^

^1^ Department of Chemistry, Faculty of Science, Universiti Putra Malaysia, 43400 UPM Serdang, Selangor Darul Ehsan, Malaysia.

^2^ Materials Synthesis and Characterization Laboratory, Institute of Advanced Technology, Universiti Putra Malaysia, 43400 UPM Serdang, Selangor Darul Ehsan, Malaysia.

^3^ Wireless and Photonics Network Research Centre, Faculty of Engineering, Universiti Putra Malaysia, 43400 UPM Serdang, Selangor Darul Ehsan, Malaysia

^4^ New Energy Science & Engineering Programme, University of Xiamen Malaysia, Jalan SunSuria, Bandar SunSuria, 43900 Sepang, Selangor Darul Ehsan, Malaysia.

*Correspondence: [janetlimhn@gmail.com](mailto:janetlimhn@gmail.com) (Hong Ngee Lim), huangnayming@xmu.edu.my (Nay Ming Huang)


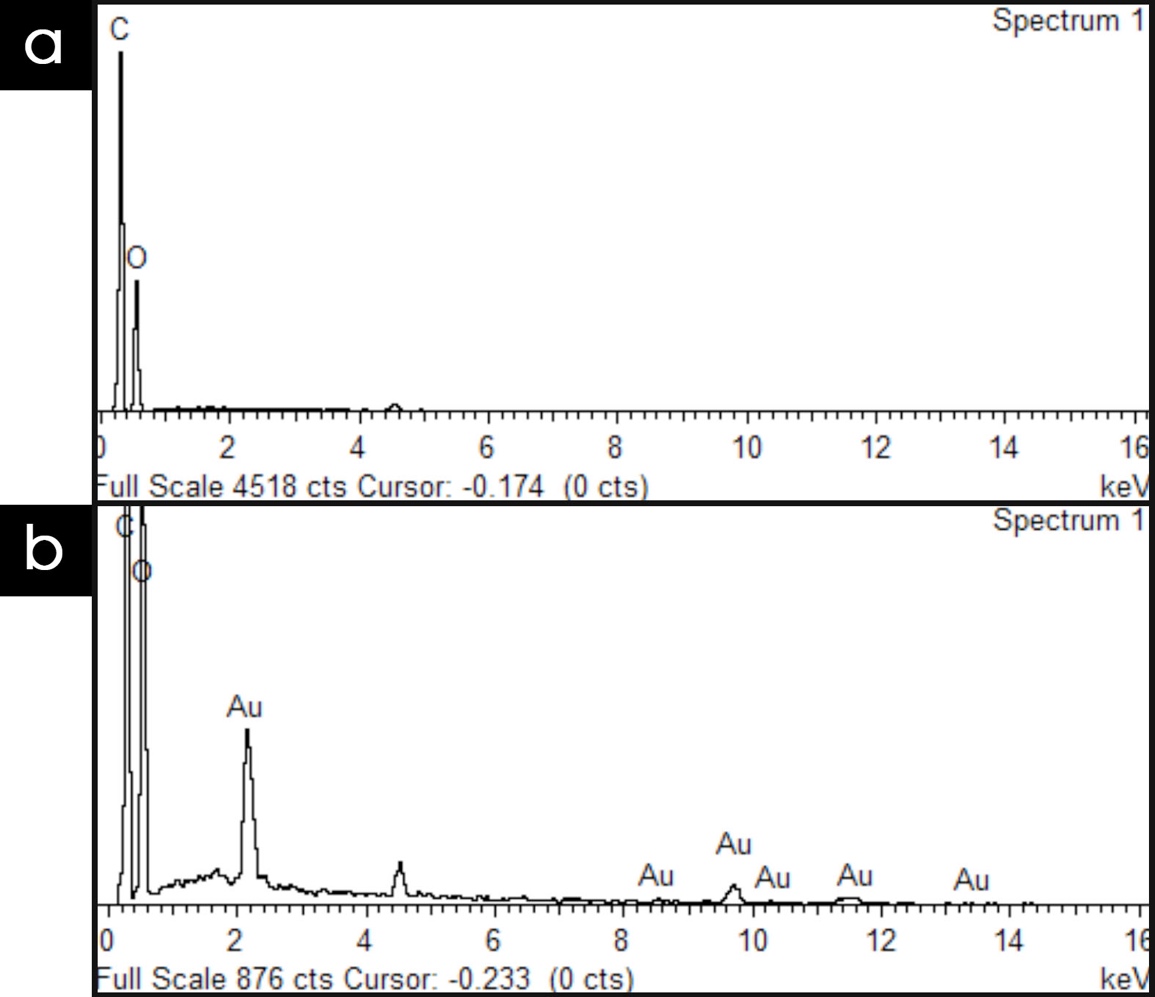


Figure S1. EDX spectra of (a) 3DE and (b) 3DE/Au electrode.


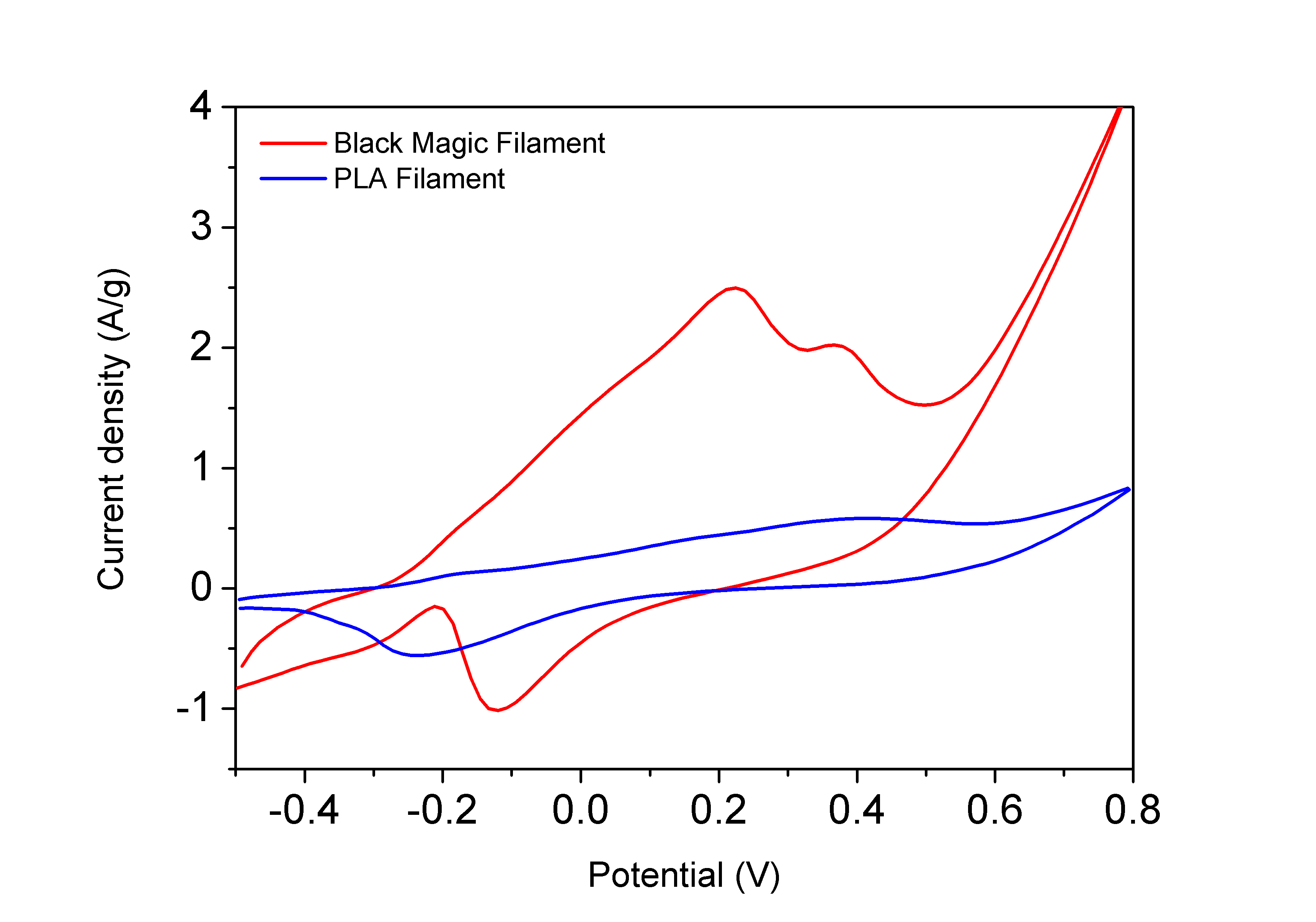


Figure S2. CV profile of 3DE made with Black Magic Filament and PLA filament, respectively.


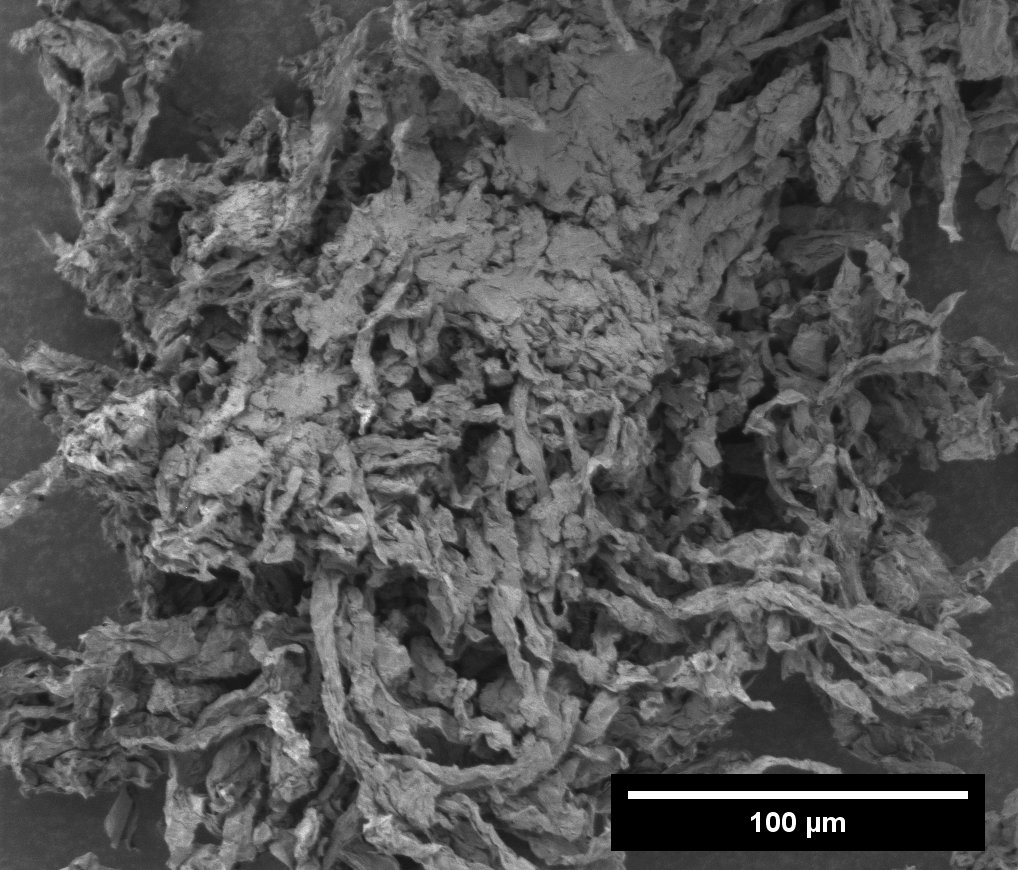


Figure S3. FESEM image of Ppy/rGO layer on 3DE/Au electrode after 1000 charge-discharge cycles.

Figure S4. Time-based photocurrent responses of 3DE/Au- and 3DE-based PEC sensors


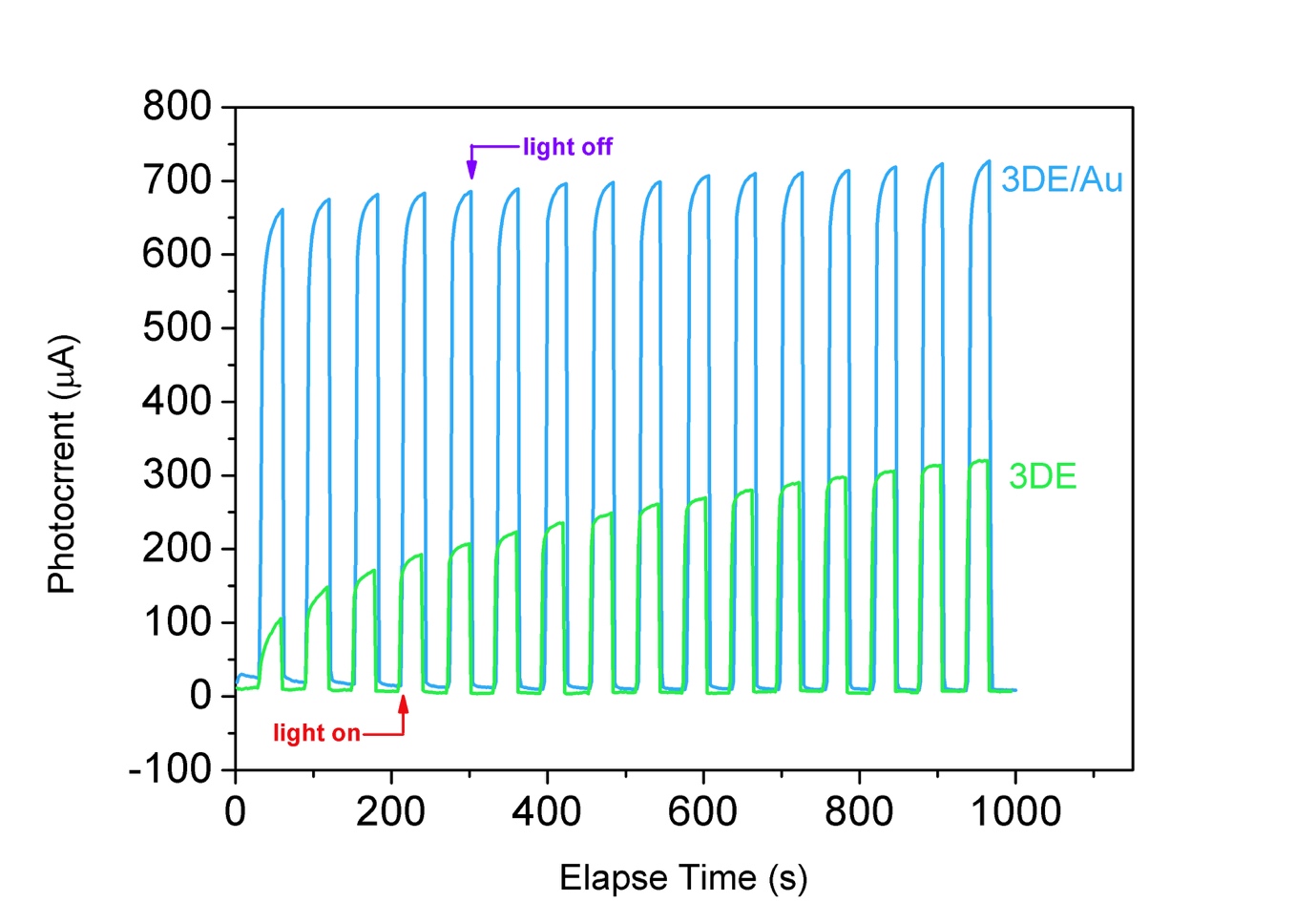


measured in KCl:TEA electrolyte at bias potential of 0.1 V.
